# Supplementary material for: STEF/TIAM2-mediated Rac1 activity at the nuclear envelope regulates the perinuclear actin cap
Source: Nat Commun. 2018 May 29;9:2124. doi: 10.1038/s41467-018-04404-4 (PMC5974301; doi:10.1038/s41467-018-04404-4)
Supplement: Supplementary file 1 — Supplementary Information [file 41467_2018_4404_MOESM1_ESM.pdf]

Supplementary Information

“STEF/TIAM2-mediated Rac1 activity at the nuclear envelope regulates the perinuclear actin cap”

Woroniuk et al

Supplementary Figure 1

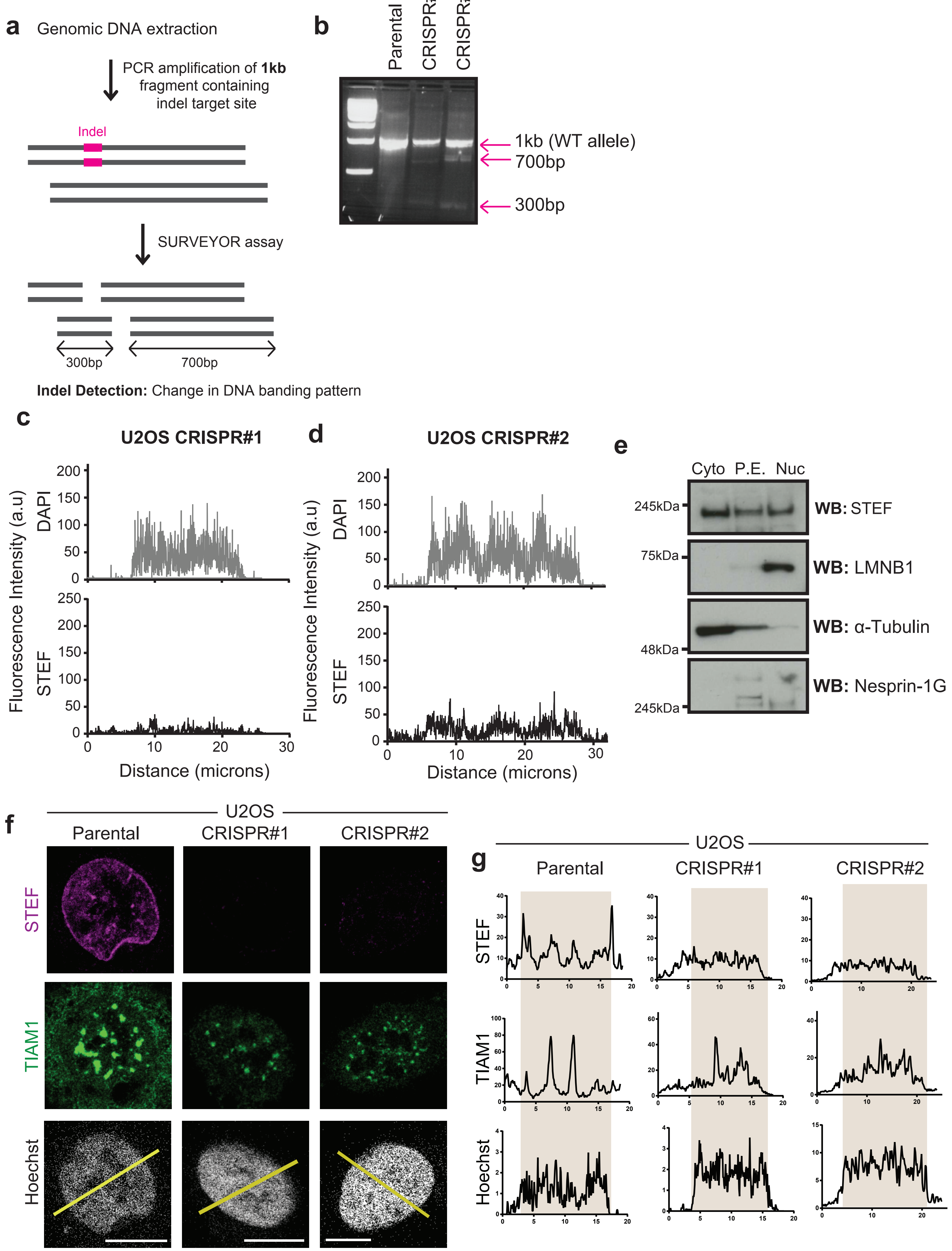

Supplementary Figure 1
STEF localises at the nuclear envelope but TIAM1 does not
a Schematic representation of the SURVEYOR assay used to measure indel formation in the targeted *STEF* locus. **b** SURVEYOR analysis of genomic DNA from two selected U2OS CRISPR clones (CRISPR #1 and #2) and parental U2OS cells. DNA banding patterns detected relative to a 1 kb DNA ladder. **c, d**) Fluorescence intensity profiles of DAPI and STEF signal across the nuclei of U2OS CRISPR#1 (**c**) or U2OS CRISPR#2 (**d**) cells. Position of line scan indicated by the dashed white line in (**a**). **e** MEFs were fractionated into cytoplasmic (Cyto), perinuclear-enriched (P.E.) and core-nuclear (Nuc) extracts using a successive lysis protocol. Lysates from each fraction were prepared containing equal total protein, and probed for expression levels of STEF alongside Tubulin (as a marker of the cytoplasmic fraction), Lamin B1 (LMNB1, as a marker of the core-nuclear fraction) and Nesprin-1G (as a marker of the perinuclear-enriched fraction). **f** Representative confocal images from one of three independent experiments of parental U2OS cells and two U2OS STEF CRISPR clones (CRISPR #1 and #2) stained for DNA (DAPI), TIAM1 and STEF. Scale bar = 10 µm. **g** Fluorescence intensity profiles of Hoechst, TIAM1 and STEF signal across the nuclei of cells shown in (**f**). Tan box indicates the position of the nucleus. Position of line scan indicated by the yellow line in (**f**).

Supplementary Figure 2

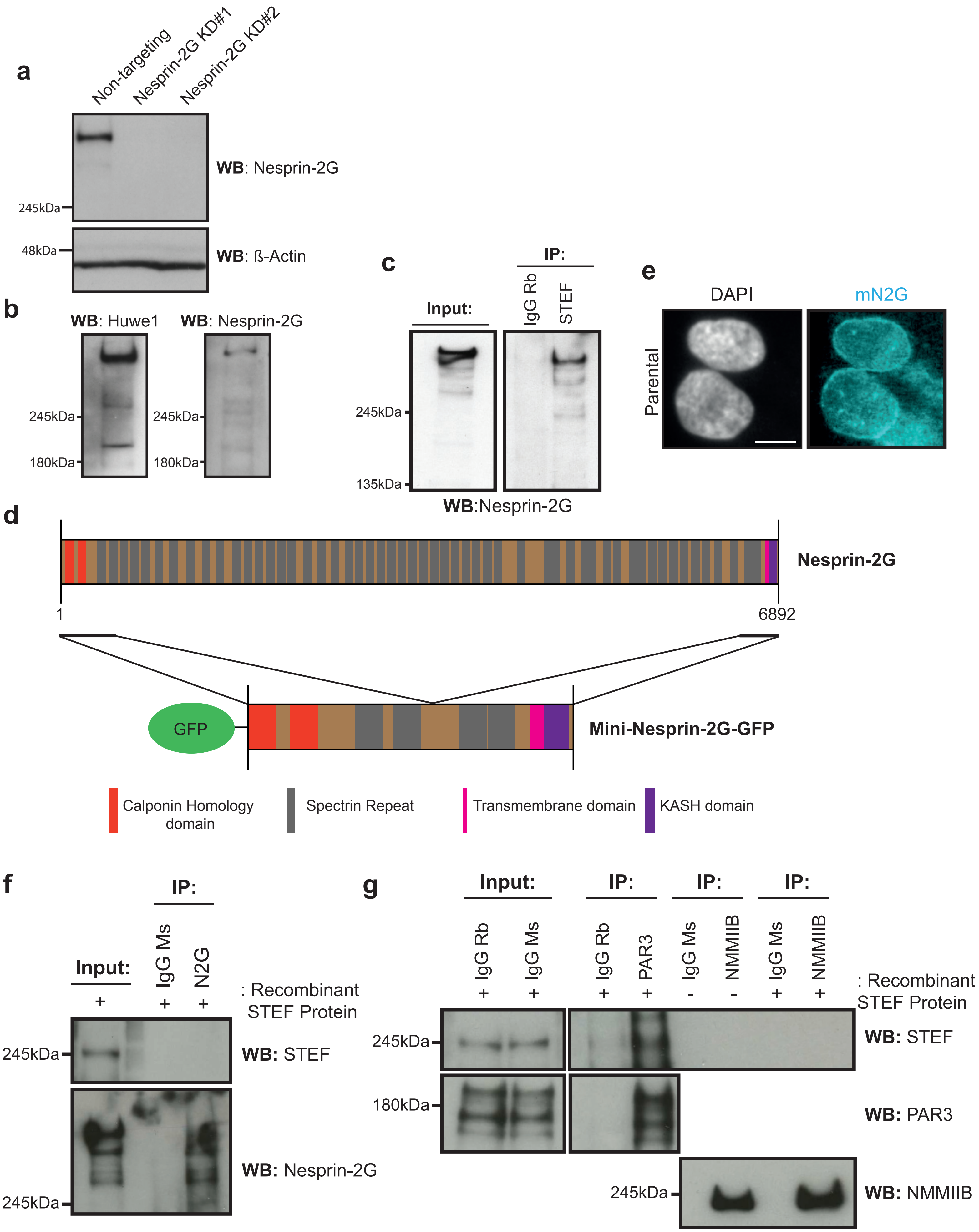

Supplementary Figure 2
STEF interacts indirectly with Nesprin-2G and NMMIIB

a Western blot showing knockdown of Nesprin-2G with two distinct siRNAs. **b** Western blot comparing the position of Nesprin-2G (796kDa) with Huwe1 (482 kDa). **c** Co-immunoprecipitation (IP) of endogenous Nesprin-2G with endogenous STEF from parental U2OS cell lysates, compared with the IgG control. Representative western blot from three independent immunoprecipitation experiments. **d** Schematic representation of the mini-Nesprin-2G-GFP construct in comparison to the endogenous giant-form, Nesprin-2G. **e** Widefield immunofluorescence images of parental U2OS cells, 48 hours after transfection with the mN2G construct. Scale bar = 10 µm. DAPI used to stain DNA and GFP to reveal localisation of mN2G. **f** Immunoprecipitated Nesprin-2G from U2OS cell lysates was incubated with purified, recombinant STEF protein in an *in vitro* pull-down assay. **g** Immunoprecipitated NMMIIB from U2OS cell lysates was incubated with purified, recombinant STEF protein in an *in vitro* pull-down assay. Immunoprecipitated PAR3 was used as a positive control.

Supplementary Figure 3

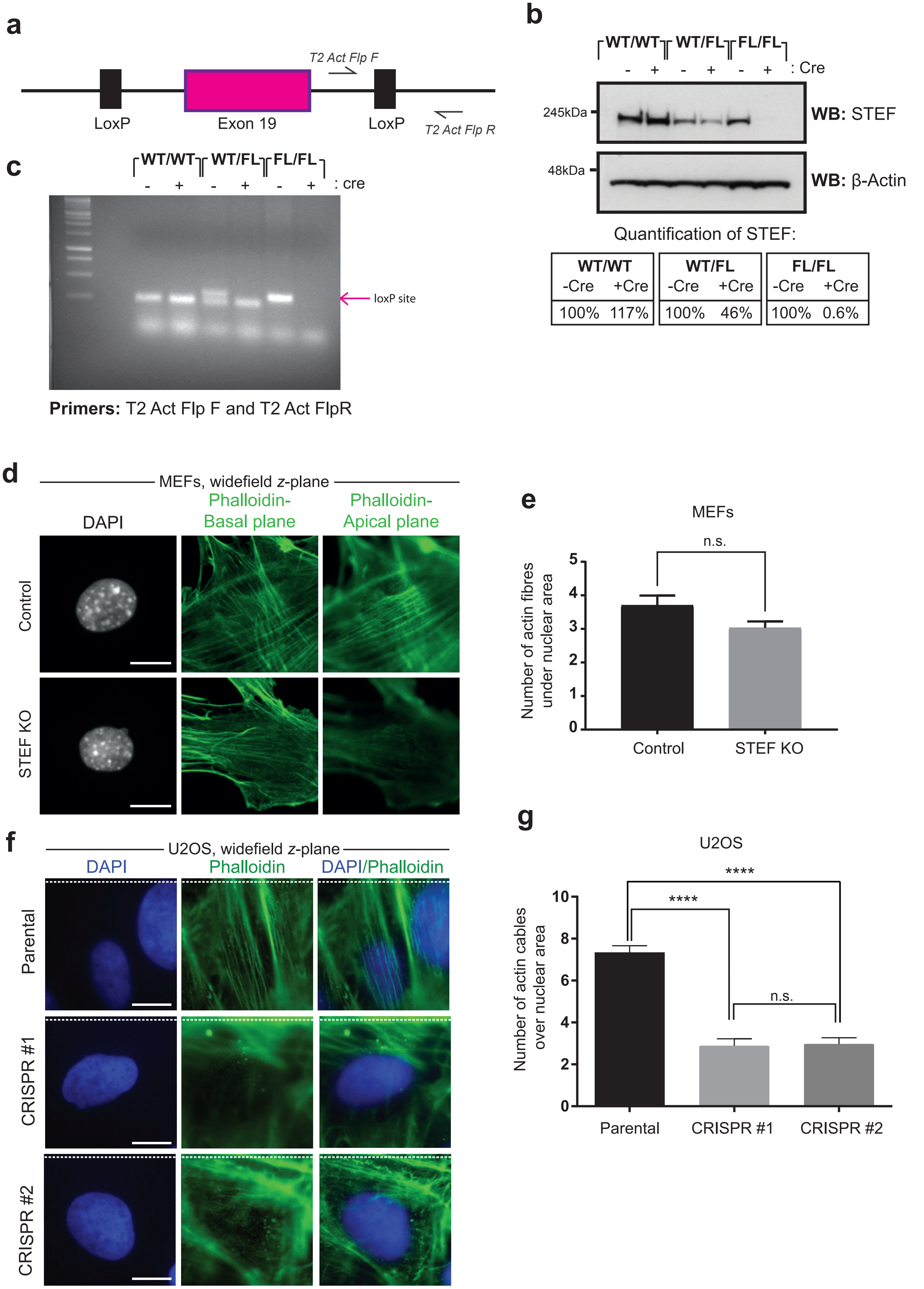

Supplementary Figure 3

STEF depletion leads to the disruption of apical perinuclear actin cables

a Schematic representation of the *stef* flox/flox locus, with the positioning of primers used for genomic PCR. **b** Western blot analysis of STEF expression levels in MEFs of different genotypes (WT/WT, WT/FL and FL/FL), with quantification from three independent experiments. MEFs were infected with either adenoviral-Cre-GFP (+ Cre) or an adenoviral-GFP control (- Cre) for 72 hours before lysates were harvested. β-actin was used as a loading control. **c** Gel electrophoresis of genomic PCR products from MEFs of different genotypes, conducted with the T2 Act Flp F/T2 Act Flp R primer pair to amplify the distal loxP site. **d** Representative widefield immunofluorescence images from one of three independent experiments of sparsely plated control and STEF KO MEFs stained for DNA (DAPI) and F-actin (Phalloidin). Two confocal *z* planes of the Phalloidin image stack are included to represent both the basal (middle column) and apical (right column) actin pools**. e** Quantification of basal actin cable number under the nuclear area from cells as in (**d**). Values represent the mean of three independent experiments (>25 cells per condition, per replicate). Statistical significance was verified using a paired, two-tailed Student's t-test. n.s. = not significant. **f** Representative widefield immunofluorescence images from one of three independent experiments of LPA-stimulated wounding assays conducted in parental U2OS and U2OS CRISPR clones, stained for DNA (DAPI) and F-actin (Phalloidin). Horizontal white line indicates the position of wounding. **g** Quantification of apical actin cable number over nuclear area in wounding assays shown in (**f**). Values represent the mean of three independent experiments (>50 cells per condition, per replicate). Statistical significance was verified using a one-way ANOVA, using Tukey's multiple comparison test to compare the means of each sample. **** p <0.0001, n.s. = not significant. Scale bars = 10 µm throughout.

Supplementary Figure 4

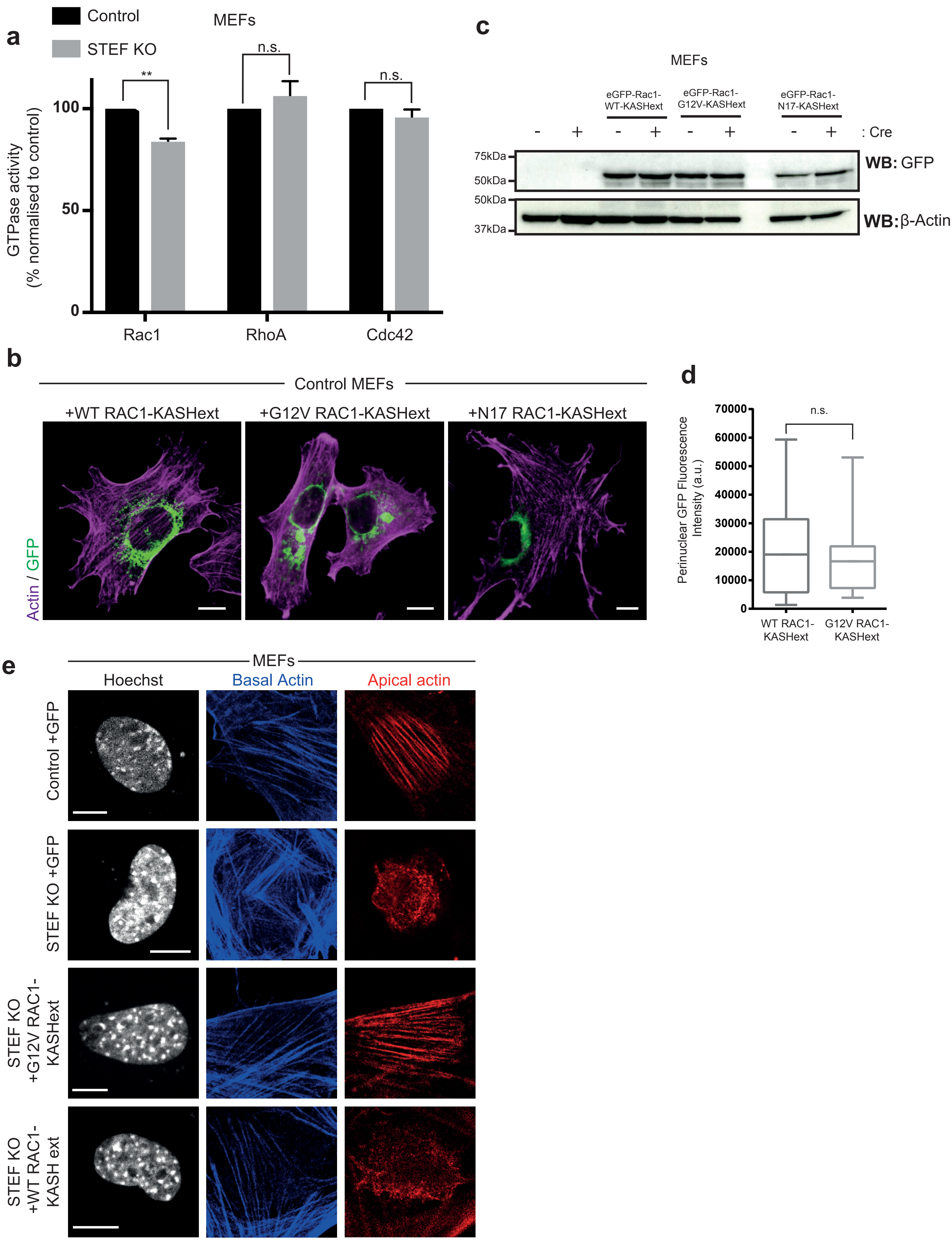

Supplementary Figure 4

Localised Rac1 activity at the nuclear envelope regulates the actin cap

a G-LISA activity assays for Rac1, RhoA and Cdc42 in control and STEF KO MEFs. Values (normalised to the control) represent the mean of three independent experiments. Statistical significance was verified using a paired t-test for each GTPase, ** $p < 0.01$, n.s. = not significant. **b** Widefield images showing localisation of WT- G12V- and N17-Rac1-KASHext constructs to the nuclear membrane, with some localisation observed in the endoplasmic reticulum. **c** Western blot showing expression of eGFP-WT-Rac1-KASHext, eGFP-G12V-Rac1-KASHext and eGFP-N17-Rac1-KASHext. **d** Box and whisker plot of perinuclear GFP fluorescence in STEF KO MEFS, expressing either the eGFP-WT-Rac1-KASHext or eGFP-G12V-Rac1-KASHext (from cells imaged for Fig. 4**d, e**). Whiskers represent min and max values, line inside the box represents the mean (>10 cells per condition, per replicate). Statistical significance was verified using a paired t-test, n.s. = not significant. **e** Representative immunofluorescence images from one of three independent experiments of control MEFs expressing a GFP only vector (top row) and STEF KO MEFs expressing either a GFP only vector (second row), nuclear envelope localised constitutively active Rac1, eGFP-G12V-Rac1-KASHext (third row), or nuclear envelope localised WT Rac1, eGFP-WT-Rac1-KASHext (fourth row). All MEFs are stained for DNA (Hoechst) and F-actin (Phalloidin), with images of both basal actin (middle column) and apical actin (right column). Scale bars = 10 µm.

Supplementary Figure 5

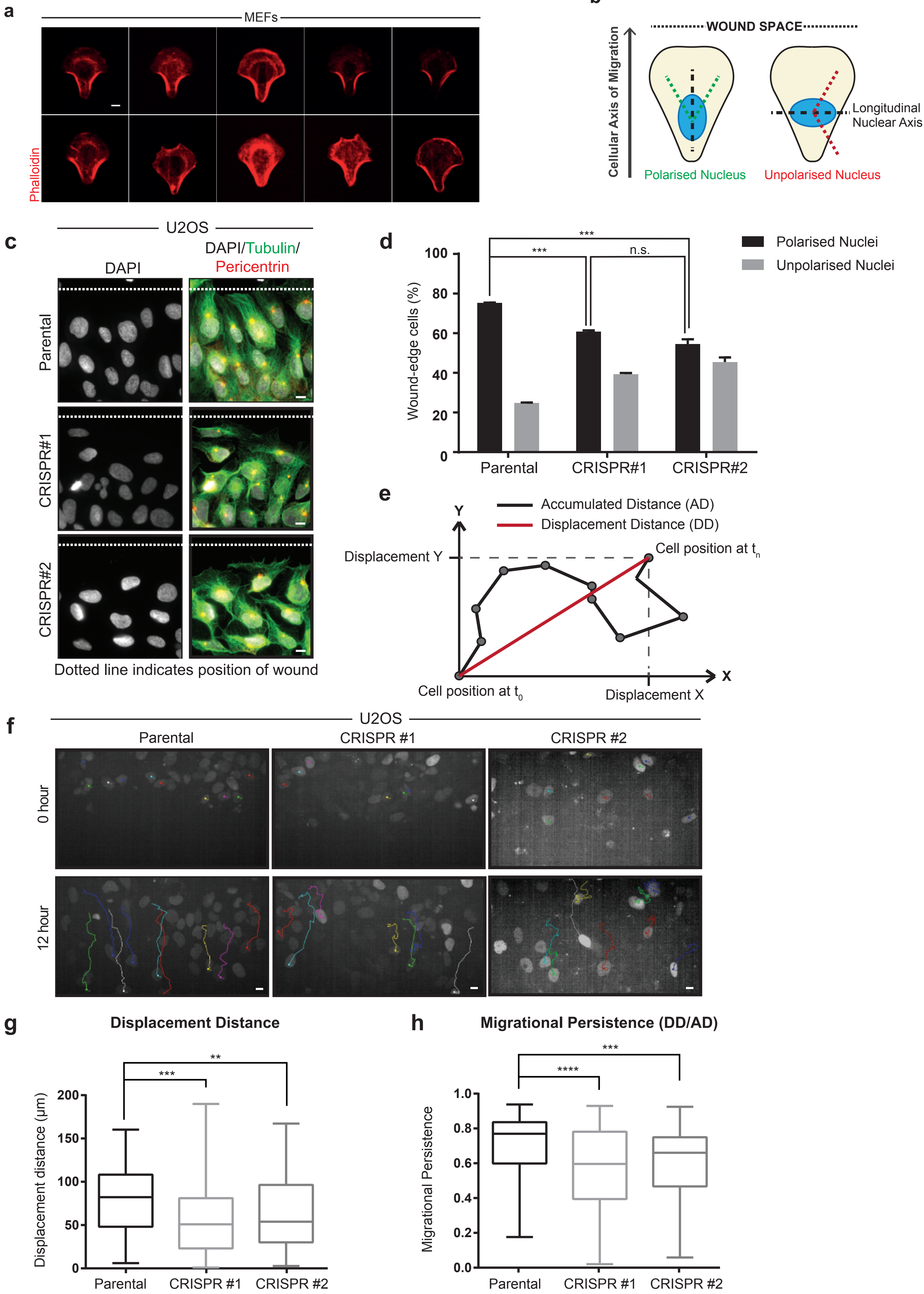

Supplementary Figure 5

STEF is required for efficient nuclear re-orientation and directed cell migration in U2OS cells

a Representative spinning disc confocal immunofluorescence images from one of two independent experiments of control MEFs plated on collagen-coated cross-bow micropatterns, fixed after 7 hours and stained for nuclei (DRAQ5) and F-actin (Phalloidin). **b** Schematic representation of the method used for scoring nuclear re-orientation in cells following wounding. **c** Immunofluorescence images of wounding assays conducted in U2OS parental and U2OS CRISPR clones, stained for DNA (DAPI), α-Tubulin (green) and the centrosomal marker Pericentrin (red) with a merged overlay. White dotted lines mark the position of wounding. **d** Quantification of nuclear re-orientation of cells in (**c**). Values represent the mean of three independent experiments. Statistical significance was verified using a one-way ANOVA, using Tukey's multiple comparison test to compare the means of each sample. *** p <0.001, n.s. =not significant. **e** Schematic representation of an example migration track, highlighting the parameters of accumulated distance and displacement distance. **f** Widefield immunofluorescence images of wounding assays of parental U2OS and U2OS CRISPR clones expressing histone-H2B-CFP. Upper panels represent cell position at 0 hours, and lower panels represent cell position at 12 hours, with colour overlay of to highlight cell tracks. Cell tracking completed using 'Manual Tracking' ImageJ plugin. **g** Quantification of displacement distance over 12 hours calculated with a vector based measurement using initial and end-point cell position. **h** Quantification of track straightness (migrational persistence) over 12 hours calculated from displacement/accumulated distance. Values represent n>120 tracks, pooled from three independent experiments. Statistical significance was verified using a one-way ANOVA, using Tukey's multiple comparison test to compare the means of each sample. ** p <0.01, *** p <0.001, **** p <0.0001. Scale bars = 10 µm throughout.

Supplementary Figure 6

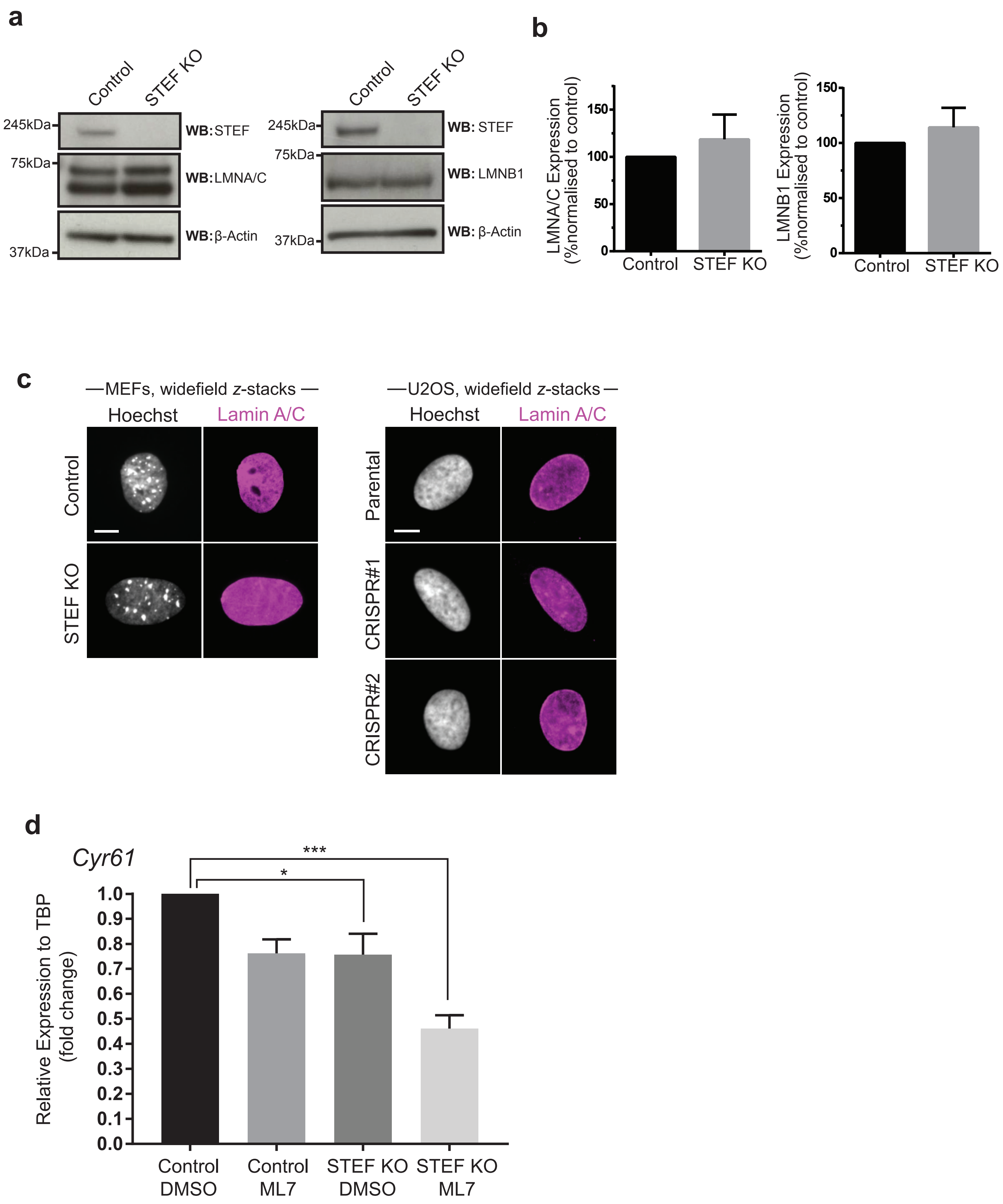

Supplementary Figure 6

The effect of STEF depletion on nuclear stiffness is not a result of aberrant lamin expression

a Western blot analysis of Lamin A/C (LMNA/C) and Lamin B1 (LMNB1) expression levels in control and STEF-depleted MEFs. β-actin was used as a loading control. Western blots are representative of five independent replicates. **b** Quantification of (**a**) from five independent experiments, normalised to the control. Statistical significance was verified using unpaired t-tests, n.s. = not significant. **c** Widefield images of Lamin A/C from control and STEF KO MEFs, and parental and CRISPR#1 and CRISPR#2 U2OS cells. **d** qPCR for the TAZ target gene *Cyr61*, normalised to *Tbp* expression in control and STEF KO MEFs pre-treated with either DMSO or 10 µM ML7 for 2 hours. Data are relative to control MEFs +DMSO and presented as mean ± S.E.M. from four independent replicates. Statistical significance was verified using a one-way ANOVA, using Tukey's multiple comparison test to compare the means of each sample. * $p < 0.05$, *** $p < 0.001$.

Supplementary Figure 7

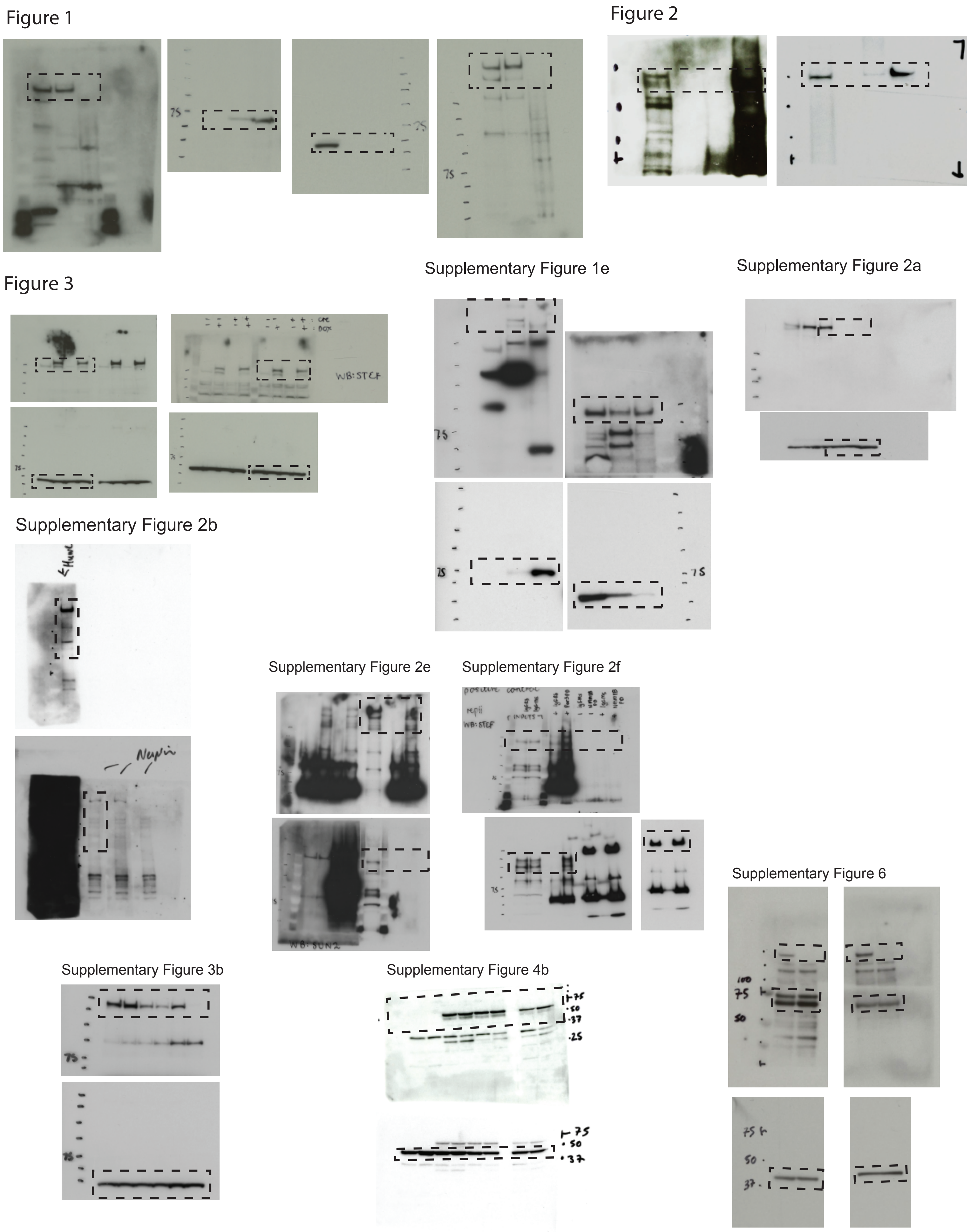

Supplementary Figure 7

Uncropped images of all western blots presented in the main and supplementary figures

Supplementary Table 1

A list of the expression vectors used or generated for the project is outlined below:

Construct Label	Vector Backbone	Insert	Supplier
FL-STEF-FLAG	pcDNA3.1	FL-STEF (mouse) with a C-terminal FLAG tag	Kind gift from Hoshino lab, National Institute of Neuroscience, NCNP
ΔN-STEF-HA	pcDNA3.1	ΔN-STEF (mouse) with a C-terminal HA tag	The Malliri Lab, CRUK MI
ΔN- ΔPHn-STEF-HA	pcDNA3.1	ΔN-ΔPHn-STEF (mouse) with a C-terminal HA tag	Kind gift from Hoshino lab, National Institute of Neuroscience, NCNP
ΔN-ΔPDZ-STEF-HA	pcDNA3.1	ΔN-ΔPDZ-STEF (mouse) with a C-terminal HA tag	Kind gift from Hoshino lab, National Institute of Neuroscience, NCNP
ΔN-ΔCC-EX-STEF-HA	pcDNA3.1	ΔN-ΔCC-EX-STEF (mouse) with a C-terminal HA tag	Kind gift from Hoshino lab, National Institute of Neuroscience, NCNP
ΔN-ΔDH-STEF-HA	pcDNA3.1	ΔN-ΔDH-STEF (mouse) with a C-terminal HA tag	Kind gift from Hoshino lab, National Institute of Neuroscience, NCNP
ΔN-ΔPHc-STEF-HA	pcDNA3.1	ΔN-ΔPHc-STEF (mouse) with a C-terminal HA tag	Kind gift from Hoshino lab, National Institute of Neuroscience, NCNP
FL-STEF-HALO	pRetroX-Tight-Pur	FL-STEF (human) with a C terminal HALO tag	Cloned by Aisha Payapilly, The Malliri Lab, CRUK MI
FL-STEF-DH*-HALO	pRetroX-Tight-Pur	FL-GEF-dead-STEF (human) with a C terminal HALO tag	Cloned by Gavin White, The Malliri Lab, CRUK MI
Histone-H2B-CFP	pBos	Histone-H2B with a CFP tag	Kind gift from Georges Laucaud, CRUK MI
mN2G-GFP	pEGFP-C1	Mini-Nesprin 2G with a C-terminal GFP tag	Dr Tobias Zech, University of Liverpool
eGFP-Rac1-WT-KASHext	pcDNA3.1	KASHext domain from Nesprin2G fused to eGFP-Rac1-WT	Cloned by Gavin White, The Malliri Lab, CRUK MI
eGFP-Rac1-G12V-KASHext	pcDNA3.1	KASHext domain from Nesprin2G fused to eGFP-Rac1, mutated to V12 (active) by site-directed mutatgenesis	Cloned by Gavin White, The Malliri Lab, CRUK MI
eGFP-Rac1-N17-KASHext	pcDNA3.1	KASHext domain from Nesprin2G fused to eGFP-Rac1, mutated to N17 (dominant negative) by site-directed mutatgenesis	Cloned by Gavin White, The Malliri Lab, CRUK MI
Raichu-Rac1-KRas-CFP-YFP			Kind gift from Pat Caswell, University of Manchester

Supplementary Table 2

List of primary and secondary antibodies and fluorescent stains used.

**Antibody**	**Dilution**	**Species**	**Distributor**
β-actin	1:5000 IB	Mouse	Sigma Aldrich
α-Tubulin	1:2000 IF<br>1:5000 IB	Mouse	Abcam ab7291 (DM1A)
Nesprin-1	1:2000 IB	Mouse	Immuquest IQ568
Nesprin-2	1:500 IF<br>1:2000 IB	Mouse	Immuquest IQ562
STEF	1:100 IF<br>1:1000 IB	Rabbit	In-house antibody
TIAM1	1:100 IF<br>1:1000 IB	Sheep	R&D Systems AF5038
LaminB1	1:1000 IB	Rabbit	Cell Signalling
Lamin A/C	1:100 IF<br>1:1000 IB	Mouse	Abcam ab8984
Non-muscle myosin IIB	1:100 IF<br>1:1000 IB	Mouse	Abcam ab684
pMLC	1:200 IF	Mouse	Cell Signalling #3675
Rac1	1:1000 IB	Mouse	BD biosciences 610650
TAZ	1:100 IF<br>1:1000 IB	Mouse	BD Biosciences 560235
Pericentrin	1:4000 IF	Rabbit	Abcam ab4448
Anti-HA	1:250 IF	Rabbit	Roche 12CA5
GFP	1:200 IF	Mouse	Roche #11814460001 (purchased from Sigma)
GFP	1:1000 IB	Rabbit	Abcam, ab290
Huwe1	1:2000 IB	Rabbit	Bethyl A300-486A
Actin	1:200 IF		Molecular Probes Alexa Fluor® 488/568 Phalloidin
Actin	1:200 IF		Texas Red Conjugated Phallodin Invitrogen T7471
DRAQ5™	1:5000 IF		Thermo Fisher Scientific
Hoechst 33342	1:2000 IF		Thermo Fisher Scientific H3570
Horseradish peroxidase (HRP)-conjugated anti-Mouse IgG	1:5000 IB		GE Healthcare
HRP-conjugated anti-Rabbit IgG	1:5000 IB		GE Healthcare
Alexa Fluor 488, 568, 647-conjugated anti-Mouse, anti-Sheep and anti-Rabbit IgG	1:500 IF		Molecular Probes
GFP-Booster_Atto488	1:200 IF		Chromotek

Supplementary Table 3

List of primers used.

**Primer Name**	**Sequence**
T2ActFlpR	AGAAACCTCTCCACGTGCTC
T2 Act Flp F	AGCCTTGGATGAGTGTAGAG
SURVEYOR FWD	GGAATGTAATTTAGGCCACGGTCTT
SURVEYOR REV	CCTGAGGGGGAGAGGCTCCCCAGGT
CRISPR sgRNA FOR	CACCGCCACCGAGTCTCGATGCGTA
CRISPR sgRNA REV	AAACTACGCATCGAGACTCGGTGGC
U6	GACTATCATATGCTTACCGT
CTGF-L	TGA CCT GGA GGA AAA CAT TAA GA
CTGF-R	AGC CCT GTA TGT CTT CAC ACT G
CYR61-L	TGA CCT CCT CGG ACT CGA T
CYR61-R	GGT TCG GTG CCA AAG ACA
TBP left	TAT TGG AGC AGA CCA TGT GC
TBP right	CAT CCT GAT TGT CAG GTG GA
**siRNA Name**	**siRNA sequence**
Nesprin2 KD#1	AAGAGAAAGAUUCUUUAGGCAACUU
Nesprin2 KD#2	AAGCAGCAGGAAGCAAAGUUUCAAC
